# Deciphering *in silico* the Role of Mutated Na_*V*_1.1 Sodium Channels in Enhancing Trigeminal Nociception in Familial Hemiplegic Migraine Type 3

**DOI:** 10.3389/fncel.2021.644047

**Published:** 2021-05-31

**Authors:** Alina Suleimanova, Max Talanov, Arn M. J. M. van den Maagdenberg, Rashid Giniatullin

**Affiliations:** ^1^Institute of Information Technology and Intelligent Systems, Kazan Federal University, Kazan, Russia; ^2^Department of Neurology, Leiden University Medical Center, Leiden, Netherlands; ^3^Department of Human Genetics, Leiden University Medical Center, Leiden, Netherlands; ^4^Laboratory of Neurobiology, Kazan Federal University, Kazan, Russia; ^5^A.I. Virtanen Institute for Molecular Sciences, University of Eastern Finland, Kuopio, Finland

**Keywords:** migraine, Na_V_, meninges, trigeminal nerve, ATP, 5-HT, FHM3, model

## Abstract

Familial hemiplegic migraine type 3 (FHM3) is caused by gain-of-function mutations in the *SCN1A* gene that encodes the α1 subunit of voltage-gated Na_V_1.1 sodium channels. The high level of expression of Na_V_1.1 channels in peripheral trigeminal neurons may lead to abnormal nociceptive signaling thus contributing to migraine pain. Na_V_1.1 dysfunction is relevant also for other neurological disorders, foremost epilepsy and stroke that are comorbid with migraine. Here we used computer modeling to test the functional role of FHM3-mutated Na_V_1.1 channels in mechanisms of trigeminal pain. The activation of Aδ-fibers was studied for two algogens, ATP and 5-HT, operating through P2X3 and 5-HT3 receptors, respectively, at trigeminal nerve terminals. In WT Aδ-fibers of meningeal afferents, Na_V_1.1 channels efficiently participate in spike generation induced by ATP and 5-HT supported by Na_V_1.6 channels. Of the various FHM3 mutations tested, the L263V missense mutation, with a longer activation state and lower activation voltage, resulted in the most pronounced spiking activity. In contrast, mutations that result in a loss of Na_V_1.1 function largely reduced firing of trigeminal nerve fibers. The combined activation of P2X3 and 5-HT3 receptors and branching of nerve fibers resulted in very prolonged and high-frequency spiking activity in the mutants compared to WT. We identified, *in silico*, key determinants of long-lasting nociceptive activity in FHM3-mutated Aδ-fibers that naturally express P2X3 and 5-HT3 receptors and suggest mutant-specific correction options. Modeled trigeminal nerve firing was significantly higher for FHM3 mutations, compared to WT, suggesting that pronounced nociceptive signaling may contribute to migraine pain.

## Introduction

The generation of disabling migraine pain involves the activation of the meningeal trigeminovascular system (Moskowitz, 2008; Messlinger, 2009), but the underlying pro-nociceptive mechanisms remain largely unknown. Current data suggest participation of mainly Aδ-fibers of the trigeminal nerve densely innervating the meninges (Melo-Carrillo et al., 2017; Haanes and Edvinsson, 2019).

To unravel molecular mechanisms of migraine pathophysiology, the study of monogenic subtypes of migraine, foremost familial hemiplegic migraine, has been instrumental (Ferrari et al., 2015). Familiar hemiplegic migraine type 3 (FHM3), which is caused by specific missense mutations in the *SCN1A* gene encoding the α1 subunit of voltage-gated Na_V_1.1 sodium channels (Dichgans et al., 2005; Tolner et al., 2015), allows the specific interrogation of the role mutant Na_V_1.1 channels in migraine pathophysiology. The observation that incubation at lower temperature and expression in neurons rescued folding/trafficking issues now firmly established that FHM3 is caused by a gain of Na_V_1.1 function (Dhifallah et al., 2018), whereas earlier studies suggested foremost loss-of-function effects of FHM3 mutations when overexpressed in heterologous expression systems (Dichgans et al., 2005; Kahlig et al., 2008). As Na_V_1.1 channels are strongly expressed in peripheral Aδ-fibers of the trigeminal nerve (Ho and O’Leary, 2011; Osteen et al., 2016), their modified activity may underlie the activation of peripheral trigeminal neurons leading to migraine pain. It can be expected that a gain-of-function enhances excitability of peripheral nerve fibers expressing modified Na_V_1.1 channels, providing a high pro-nociceptive activity delivered to second order neurons.

Investigating Na_V_1.1 channel dysfunction, which are expressed also in central neurons (Ogiwara et al., 2007; Favero et al., 2018; Sakkaki et al., 2020), in relation to changes in neuronal excitability, is also of relevance to various neurological disorders other than migraine, foremost childhood epilepsy (Menezes et al., 2020), autism spectrum disorder (Scheffer and Nabbout, 2019), Alzheimer’s disease (Sakkaki et al., 2020), and perhaps less known, transient cerebral ischemia (Zhan et al., 2007). In fact, changes in neuronal hyperexcitability may therefore, at least to certain extent, underlie the comorbidity of several of the disorders with migraine, for instance when they lead to spreading depolarizations, as observed for migraine and stroke (Dreier and Reiffurth, 2015). Unlike for the other disorders, the evidence for a specific role of Na_V_1.1 channels in cerebral ischemia is limited although voltage-gated cation channels, including sodium channels have been targets for the treatment of stroke (Gribkoff and Winquist, 2005).

The predominant hypothesis for triggering peripheral mechanisms of pain is that trigeminal nerve terminals are activated by local depolarizing stimuli (Julius and Basbaum, 2001; Basbaum, 2002; Giniatullin, 2020). Purinergic and serotonergic mechanisms are among the most powerful triggers of peripheral nociception in meningeal afferents (Yegutkin et al., 2016; Kilinc et al., 2017; Koroleva et al., 2019).

Recently, we presented a mathematical model of the nociceptive neuro-immune synapse in meninges that, by activation with ATP and 5-HT, generates neuronal firing (Suleimanova et al., 2020). Meninges, which are densely innervated by trigeminal nerve fibers, are currently considered a main source of migraine headache (Moskowitz, 2008; Messlinger, 2009; Olesen et al., 2009). The two algesic substances, ATP and 5-HT selected to be modeled in this study produce a powerful and long-lasting activation of meningeal afferents (Yegutkin et al., 2016; Kilinc et al., 2017; Koroleva et al., 2019). The data are consistent with the purinergic hypothesis of migraine suggested earlier by Burnstock (1981). There is strong evidence that serotonin (5-HT) is involved in migraine already because “triptans,” serotonin (5-HT1B/1D) agonists, are effective in treating migraine patients (Goadsby, 2007). However, the sources of the two neurotransmitters, the time spent in the extracellular space, the receptor kinetics, and most notably, the speed of desensitization of the transmitters are different, as we presented in our previous model (Suleimanova et al., 2020). Notably, the respective P2X3 and 5-HT3 receptors activated by ATP and 5-HT, respectively, are expressed in Aδ-fibers (Ford, 2012; Kilinc et al., 2017; Sato et al., 2018), thus in the same neurons that express Na_V_1.1 sodium channels. Combining, in the mathematical model, effects of these triggers with dysfunction of Na_V_1.1 channels due to mutations identified in patients may serve as a useful platform to explore mechanisms of activation of peripheral nociception relevant to migraine headache.

Therefore, we here assessed, by using *in silico* modeling, how gain- and loss-of-function mutations in the α1 subunit of Na_V_1.1 channels, as they occur in patients with FHM3 and childhood epilepsy, respectively, might affect peripheral trigeminal nociception in meningeal afferents. To this end, the WT vs. various mutants Na_V_1.1 channels were *in silico* “co-expressed” along with other types of sodium (Na_V_1.6, Na_V_1.7, Na_V_1.8) and several potassium channel types (K_V_1, K_V_3, K_V_4, and calcium-activated potassium channel K_Ca_) according to published profile of these channels in Aδ-fibers (Tigerholm et al., 2014; Mandge and Manchanda, 2018; Zemel et al., 2018; Zheng et al., 2019). Our data show a large amplification of nociception in meningeal afferents exclusively with gain-of-function mutations providing a scientific framework that a peripheral mechanism of generating migraine pain in patients with FHM3.

## Materials and Methods

### The Mathematical Model for Testing of FHM3 Mutations

**FIGURE 1:**
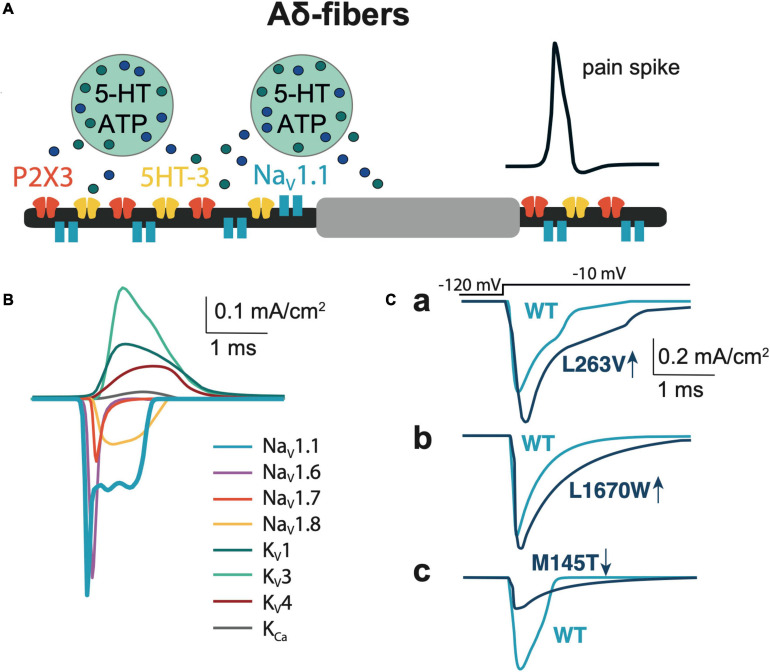
Model of Aδ-fiber. **(A)** Schematic presentation of the Aδ-fiber with the diameter 5 μm and node of Ranvier 2 × 2 μm. **(B)** The membrane Na_V_, K_V_, and K_*Ca*_ currents relevant to the model of Aδ-fiber. **(C)** Comparison of Na_V_1.1 currents, that evoked by a voltage step to -10 mV of WT with the L263V **(Ca)**, L1670W **(Cb)**, and M145T **(Cc)** mutations.

To model the function of the trigeminal nerve in meninges we used the NEURON environment version 7.8 (Hines and Carnevale, 2003). Our model describes the activity of Aδ-fibers induced by single, or repetitive ATP and 5-HT release events from abundant meningeal mast cells (Theoharides et al., 1995; Levy, 2009; Kilinc et al., 2017). Thus, the Aδ-fiber coupled to a mast cell represent a model of the nociceptive “neuro-immune synapse” (Theoharides et al., 1995; Koroleva et al., 2019; Suleimanova et al., 2020; Figure 1A). Within such synapse, locally released ATP or 5-HT activates, at nerve terminals, P2X3 or 5-HT3 receptors, respectively. Both ATP and 5-HT are potent triggers of nociceptive firing in meningeal afferents (Yegutkin et al., 2016; Kilinc et al., 2017; Koroleva et al., 2019). Although P2X2 receptors are co-expressed with P2X3 subunits in rodents (Simonetti et al., 2006), the P2X3 subtype is the predominant ATP receptor subtype in human sensory neurons (Serrano et al., 2012). Therefore, in our model, we used P2X3 receptors as the main target for fast action of ATP on trigeminal meningeal afferents.

We explored the action of different concentrations of two algogens but for most model trials we used 1 μM ATP and 2 μM 5-HT concentrations to activate receptors, since these values are close to EC_50_ of the respective receptors (Sokolova et al., 2006; Corradi et al., 2009). The kinetics of P2X3 receptors was based on the previously published model of this receptor (Sokolova et al., 2006), whereas the kinetic of 5-HT3 receptors was taken from the study of Corradi et al. (2009). The lifetime of extracellular ATP is determined by fast hydrolysis via multiple ecto-enzymes (Yegutkin, 2008), whereas the profile of 5-HT is controlled by a relatively slow uptake via SERT transporters (Wood et al., 2014). Therefore, we included in our model the partial hydrolysis for ATP and uptake for 5-HT making the model consistent with experimental data on the action of ATP and 5-HT (Suleimanova et al., 2020). The 3D diffusion model suggested by Saftenku (2005) was used to reproduce the time course of transmitters in the nociceptive synapse. We also conducted in the current model experiments in which we varied ATP and 5-HT concentrations to explore the dependence of spike firing on the profile of the neurotransmitters in the meningeal neuro-immune synapse.

The diameter of the Aδ-fiber is 5 μm (West et al., 2015), each segment of the fiber consists of a paranode and a node of Ranvier (McIntyre et al., 2002) that is 2 × 2 μm (width and length; Figure 1A). Aδ-fibers highly expressed tetrodotoxin-sensitive (TTX-sensitive) Na_V_1 channel type (Na_V_1.1, Na_V_1.6, Na_V_1.7), while Na_V_1.8 was at low level compared to C-fibers (Pinto et al., 2008; Zhang et al., 2013). Most of the input parameters on channel kinetics and voltage dependence of Na_V_1.6, K_V_1, K_V_3, and K_V_4 ion channels were obtained from somatic recordings (Zheng et al., 2019), whereas the data for the density of ion channels were obtained from axonal measurements (Waxman and Ritchie, 1993; McIntyre et al., 2002). Likewise, we used data from somatic recordings of the contribution of Na_V_1 channels to I_Na_ current (Zhang et al., 2013) and Na_V_ channels mRNA levels (Ho and O’Leary, 2011). The WT Na_V_1.1 channel and FHM3-associated mutations in this channel were modeled based on functional properties obtained in neurons or tsA201 cells (for details see Table 1).

**TABLE 1:** Biophysical parameters for activation, fast inactivation and slow inactivation of WTs and mutants of Na_V_1.1 channel.

**Channel**	**Activation (mV)**		**Fast inactivation (mV)**		**Slow inactivation (mV)**		**tau*_slow_* (ms) at −10 mV**		**References**
	**V_1__/__2_**	**k**	**V_1__/__2_**	**k**	**V_1__/__2_**	**k**	**experiment**	**model**	
**Familial hemiplegic migraine type 3 (FHM3)**									
L263V	−24.6	7.1	−54.4	6.7	−54.1	5.0	3,507	3,500	Kahlig et al., 2008
gain-of-function/WT	−21.5	7.2	−62.2	6.4	−66.8	6.3	2,100	2,100	
Q1478K combined	−24.8	9.1	−60.1	7.4	−77.2	7.7	938 (−5 mV)	940 (−5 mV)	Cestele et al., 2008
gain- and loss-of function/WT	−24.5	7.9	−65.1	6.0	−75.8	6.2	2.090 (−5 mV)	2,100 (−5 mV)	
L1649Q	−22.9	7.4	−34.5	7.1	−57.7	8.9	1,885 (0 mV)	1,885 (0 mV)	Cestele et al., 2013
gain-of-function/WT	−21.0	6.6	−54.2	5.5	−59.7	9.7	2,616 (0 mV)	2,600 (0 mV)	
L1670W	−16.8	6.1	−47.3	6.1	−34.8	10.0	2,070	2,070	Dhifallah et al., 2018
gain-of-function/WT	−21.6	6.6	−55.6	4.9	−53.5	7.6	1,310	1,300	
**Familial simple febrile seizures**									
M145T	−11.7	7.1	−65.5	9.1	–	–	–		Mantegazza et al., 2005
loss-of-function/WT	−21.7	6.15	−64.9	8.0					
**Generalized epilepsy with febrile seizures plus (GEFS +)**									
R1648H	−19.9	8.5	−61.3	7.8	−68.6	6.1	3,112	3,112	Kahlig et al., 2006
gain-of-function/WT	−19.4	7.9	−62.7	6.9	−66.7	7.8	3,029	3,000	

*V_1__/__2_ is the voltage of half-maximal activation or inactivation; k is a slope factor; tau_*slow*_ is kinetics of the development of slow inactivation. Voltage indicated in brackets shows values at which the recordings were performed in the quoted papers and in model studies.*

To match the natural profile of sodium channels in the Aδ-fiber, we revised our previously used mathematical model (Suleimanova et al., 2020) by adding Na_V_1.1 and Na_V_1.6, since these fibers express both type of these channels in nodes of Ranvier (Duflocq et al., 2008; Leterrier et al., 2011). We also added Na_V_1.7 and Na_V_1.8 channels to our model, as they also support the generation and propagation of nociceptive signals from the periphery (Duflocq et al., 2008; Black et al., 2012; Figure 1B and Supplementary Table 1). The maximum conductance densities of the TTX-sensitive channels are based on single-channel conductance and channel density values in the range of 1,000 to 2,000 channels/μm^2^ (McIntyre et al., 2002). The density of TTX-resistant Nav1.8 channel is significantly lower since TTX completely blocked spiking activity in Aδ-fiber (Pinto et al., 2008), whereas specific blockers of TTX-resistant channels did not significantly reduce spiking (Tsuchimochi et al., 2011). Of note, we tuned the values to provide a closer similarity to action potential and firing patterns observed in the experimental studies (Cestele et al., 2008, 2013) taking into account the contributions of Na_V_1.1, Na_V_1.6, and Na_V_1.7 to TTX-sensitive current (Zhang et al., 2013) and therespective Na_V_ channels mRNA level (Ho and O’Leary, 2011). When modeling, we also took into consideration a fiber-specific difference in expression of potassium channels (Zemel et al., 2018; Chien et al., 2007). Thus, our Aδ-fiber model includes voltage-gated potassium channels K_V_1, Kv3, and Kv4 mediating A-type currents, which are found in axons and nerve endings (Zemel et al., 2018). We recently showed that 4-Aminopyridine, a blocker of A-type current (determined by voltage-gated potassium Kv1 and Kv3 channels) dramatically enhanced the firing of trigeminal afferents in rat meninges promoting appearance of fast large spikes (Andreou et al., 2020), typical for the phenotype of trigeminal Aδ-fibers (MacIver and Tanelian, 1993). We also added to the model calcium-activated potassium large-conductance K_Ca_ channels (BK_Ca_) (Mandge and Manchanda, 2018). The full set of maximum conductance densities of the channels and the kinetics underlying the single action potential is shown in Supplementary Table 1 and Figure 1B, where the current time course for each mutant (L263V, L1670W, and M145T) was compared with the respective WT to fit with experimental results from Mantegazza et al. (2005); Kahlig et al. (2006), and Dhifallah et al. (2018).

Next, we modeled various *SCN1A* mutations by using steady-state voltage dependence and kinetics of channel activation and inactivation as presented in Spampanato et al. (2004). First, we fitted the kinetics of the channels using the three-parameter Gaussian exponential function: τ = a × exp (((V–V_0_)/b)^2^), where V is the membrane voltage, V_0_ is the mean voltage around which the Gaussian is positioned, a is the height, and b is the standard deviation. Second, the steady-state voltage dependence of activation was calculated by a Boltzmann function in the form: m_∞_ = 1/(1 + exp(–(V–V_1__/__2_)/k) and h_∞_/s_∞_/r_∞_ = 1/(1 + exp((V–V_1__/__2_)/k) for fast (h_∞_), slow (s_∞_) inactivation and recovery (r_∞_), where V_1__/__2_ is the voltage of half-maximal activation or inactivation, and k is a slope factor. The constants for these calculations are presented in Table 1. Third, we used the Hodgkin-Huxley formalism where activation (m) and inactivation (h) states were in the range between 0 and 1 dependent on voltage and time. We used the following equation to define the channel activation state: dm/dt = (m_∞_–m)/τ_m_, where m_∞_ is referred to as steady-state voltage dependence and τ_m_ is the kinetics. In a similar way, we determined the fast and slow inactivation states and recovery. We assume that the slow inactivation and recovery states are independent from other channel gating states since development of slow inactivation and recovery was recorded separately in experimental papers, we added *s*- and *r*-variable to the model. Thus, the sodium current is described by following equation:

$$I_{\text{Na}}=m^{3}\,h\,s\,r\,g_{\text{Na}}*\left(V-E_{\text{Na}}\right)$$

where g_Na_ is the maximum conductance; E_Na_is the equilibrium potential for sodium; *m* and *h* are the voltage-dependent activation and inactivation gating states; *s* and *r* are the additional slow inactivation development and recovery voltage-dependent variables.

**FIGURE 2:**
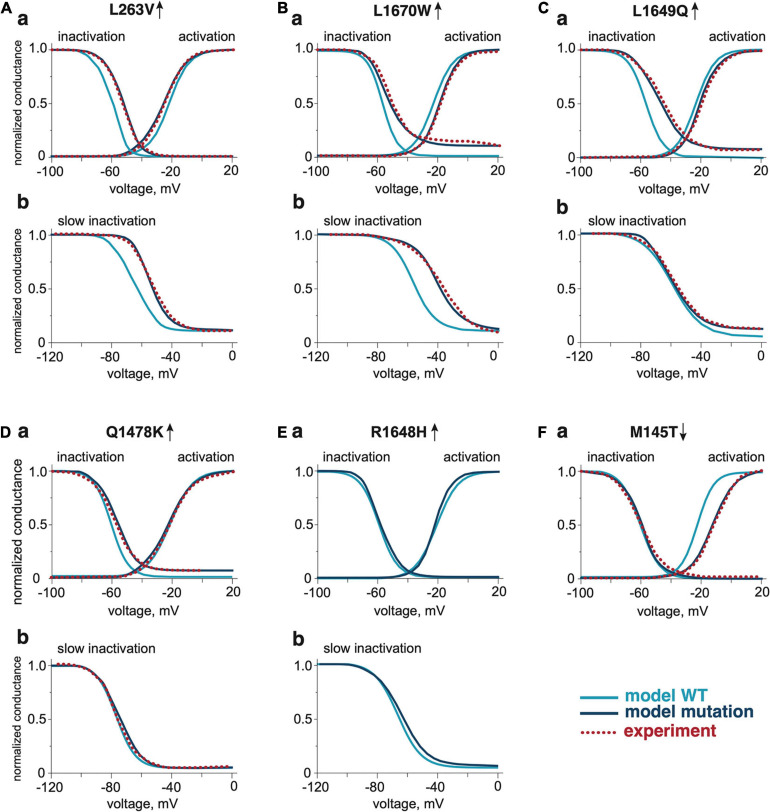
The activation and inactivation properties of WT and Na_V_1.1 mutants. The comparison of voltage dependence of activation and fast inactivation of Na_V_1.1 WT and mutants L263V **(Aa)**, L1670W **(Ba)**, L1649Q **(Ca)**, Q1478K **(Da)**, R1648H **(Ea)**, and M145T **(Fa)**. The comparison of voltage dependence of slow inactivation development of Na_V_1.1 WT and gain-of-function mutants L263V **(Ab)**, L1670W **(Bb)**, L1649Q **(Cb)**, Q1478K **(Db)**, and R1648H **(Eb)**. The dotted line shows experimental results from Kahlig et al. (2008) for the L263V mutation, Dhifallah et al. (2018) for the L1670W mutation, Cestele et al. (2008) for the L1649Q mutation, Cestele et al. (2013) for the Q1478K mutation, and Mantegazza et al. (2005) for the M145T mutation. Mutations were compared with the respective WT data from the publication that presented experimental data for the mutations.

We modified the activation voltage and the activation speed of WT Na_V_1.1 channels (Zheng et al., 2019) to recapitulate better the characteristics of a FHM3 mutation. To this end, we decreased the activation voltage of FHM3-associated Na_V_1.1 channels (V_1__/__2_ of WT = −21 mV, V_1__/__2_ of FHM3 = −25 mV; Figures 2Aa,Ab and Table 1) since L263V-mutated Na_V_1.1 (Na_V_1.1-L263V) channels are activated at a lower voltage than WT Na_V_1.1 channels, although the difference was not significant (Kahlig et al., 2008). The FHM3 L263V mutation shows slower kinetics than WT for both the activation and the inactivation state and an increased “window current” (Figure 2Aa and Supplementary Figure 2A) due to the shift of the voltage dependence of activation curve to lower voltages and the inactivation curve to higher voltages compared to WT (Kahlig et al., 2008), thus the current peak of Na_V_1.1-L263V is wide (Figure 1Ca and Supplementary Figure 3). Second, we modeled the FHM3 L1670W mutation (Figure 1Cb), which also exhibits a gain-of-function effect (Dhifallah et al., 2018). This mutation increases the persistent sodium current and shows a positive shift of the inactivation curve and faster recovery from fast inactivation in comparison to WT (Figures 2Ba,Bb and Supplementary Figures 1B, 2B). Third, the FHM3 L1649Q mutation also enhances persistent sodium current, recovery from slow inactivation, and slightly increased the time constant for fast inactivation (Figures 2Ca,Cb and Supplementary Figures 1C, 2C), which can be the reason for the prolonged spiking activity (Cestele et al., 2013). Fourth, the FHM3 Q1478K mutation shows faster recovery, a positive shift of the voltage dependence of inactivation (Figures 2Da,Db and Supplementary Figures 1D, 2D), and a higher amplitude persistent current (Cestele et al., 2008). Next, we modeled to the model the R1648H mutation (Figures 2Ea,Eb and Supplementary Figures 1E, 2E) that is associated with childhood epilepsy but exhibits a persistent sodium current as well (Kahlig et al., 2006). Finally, for comparison, we modeled the loss-of-function mutation M145T (Figures 1Cc, 2Fa). The comparison of activation and inactivation properties of the WT and mutant Na_V_1.1 channels are shown in Figure 2 and Supplementary Figures 1, 2. Using experimental data from Mantegazza et al. (2005); Kahlig et al. (2006, 2008); Cestele et al. (2008, 2013), and Dhifallah et al. (2018) for the mutant and respective WT channel characteristics, we validated our model data with experimental results describing the voltage-dependence of channel activation and inactivation by voltage steps (with increment 10 mV) from −100 to 20 mV. The time constant of development of fast inactivation are shown in Supplementary Figure 2. The distribution of tau in our model was compared with time constant from experimental results at the different potentials in the range (−65 to 30 mV). We used median values of normalized conductance at voltage from −100 to 20 mV with a 10-mV step increment. Based on these values, we plotted steady-state curves, using interpolation, and compared results with simulated voltage-dependent steady-states. This comparison indicating a high similarity (*p*-values with Kolmogorov–Smirnov test (K-S test) higher than 0.1 (Boyerinas, 2016) between experimental results and simulation data (Figure 2, notice dotted lines for experimental results).

Finally, we took into consideration that the meningeal nerve has a branched structure (Schueler et al., 2014; Barkai et al., 2020; Suleimanova et al., 2020). Therefore, we modified our model to a tree structure with two 1.1 cm long axon branches. In this version of the model, we added two varying concentrations of ATP or 5-HT to activate each of the axon branches. As the junction of branches can block spikes from a branch in the refractory state, we calculated that an interval of 15 ms between applications was sufficient for the propagation of spiking activity.

### Statistical Analysis

We used Kolmogorov–Smirnov test (K-S test) (Boyerinas, 2016) to compare the distribution of inter-spike intervals in experiments with simulation. The ks_2samp function from SciPy library that is implemented in the K-S test statistics was used to compare two samples. With this approach, the *p*-value higher than 0.05, allowed to accept the null hypothesis indicating that the distribution of data in two samples are similar. Experimental data for validation of the model were taken from our publication describing the action of ATP and 5-HT on mouse trigeminal nerves (Koroleva et al., 2019).

## Results

### Role of Na_V_1.1 and Na_V_1.6 Channels in ATP-Induced Activation of WT and FHM3 Aδ-Fibers

**FIGURE 3:**
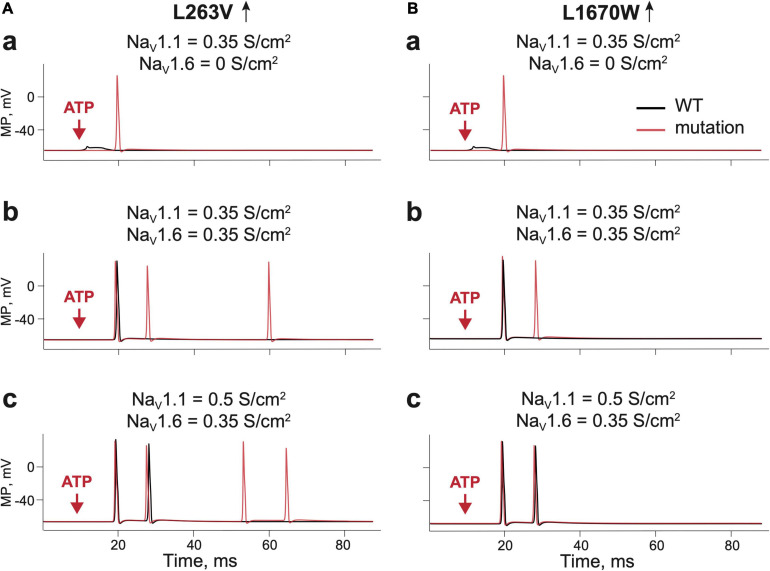
Testing the role of Na_V_ 1.1 and Na_V_1.6 in firing of Aδ-fiber with one branch and a single ATP release event. **(Aa)** The receptor potential in a single WT (Kahlig et al., 2008) fiber expressing Na_V_1.1 (black line) and firing in the fiber without Na_V_1.6. with L263V mutation (red line) of Na_V_1.1 with conductance 0.35 S/cm^2^. Of note, there is a membrane potential in the nerve compartment, where ATP is acting, whereas in other panels we present the appearance of the signal to the trigeminal ganglion. **(Ab)** WT (Kahlig et al., 2008) fiber (black line) and the L263V mutation (red line) with Na_V_1.1 and Na_V_1.6 (both with conductance 0.35 S/cm^2^) and; **(Ac)** WT (Kahlig et al., 2008) fiber (black line) and the L263V mutation (red line) with Na_V_1.1 conductance 0.5 S/cm^2^ and Na_V_1.6 conductance 0.35 S/cm^2^. **(Ba)** The receptor potential in a single WT (Dhifallah et al., 2018) fiber (black line) and L1670W mutation (red line) of Na_V_1.1 conductance 0.35 S/cm^2^ and without Na_V_1.6. **(Bb)** WT (Dhifallah et al., 2018) fiber (black line) and the L1670W mutation (red line) with Na_V_1.1 conductance 0.35 S/cm^2^ and Na_V_1.6 conductance 0.35 S/cm^2^. **(Bc)** WT (Dhifallah et al., 2018) fiber (black line) and the L1670W mutation (red line) with Na_V_1.1 conductance 0.5 S/cm^2^ and Na_V_1.6 conductance 0.35 S/cm^2^. ATP signaling is limited by partial hydrolysis.

Aδ-fibers are thought to play an important role in the generation of migraine pain (Melo-Carrillo et al., 2017; Haanes and Edvinsson, 2019). As Na_V_1.1 and Na_V_1.6 channels are the major Na_V_ channel types in Aδ-fibers, we first explored their role in ATP-induced activation of WT and FHM3 nerve fibers. We first used a simplified model of the single trigeminal nerve fiber with one release site for ATP. To address the pure Na_V_1 phenotype we started with *in silico* “knocking down” the Na_V_1.6 channel subtype in WT, whereas the conductance of Na_V_1.1 was set to 0.35 S/cm^2^. In this case, ATP only induced local receptor potential (Figures 3Aa,Ba black lines). Adding Na_V_1.6, with a conductance of 0.35 S/cm^2^ was sufficient to generate propagating spikes (Figures 3Ab,Bb black lines). Raising the activity of Na_V_1.1 (conductance 0.5 S/cm^2^) enhanced the firing with repetitive spikes (Figures 3Ac,Bc black lines). In contrast to WT, in the FHM3 model with gain-of-function mutations L263V and L1670W, even Na_V_1.1 alone was sufficiently effective to generate propagating spikes (Figures 3Aa,Ba red lines). Even higher activity was obtained when Na_V_1.6 (Figures 3Ab,Bb red lines) was added or when the conductance of the Na_V_1.1 channel was increased to 0.5 S/cm^2^ (Figures 3Ac,Bc red lines).

Thus, even in this simple linear model, the activity of FHM3 gain-of-function mutations produced an increased number of nociceptive spikes, suggesting that excitability of the terminals of meningeal afferents was increased. Notably, the L263V mutation caused more spikes than the L1670W mutation when compared with the respective WTs.

### Specific Role of Na_V_1.1 Channels in the Spiking Activity of WT and FHM3 Aδ-Fibers

**FIGURE 4:**
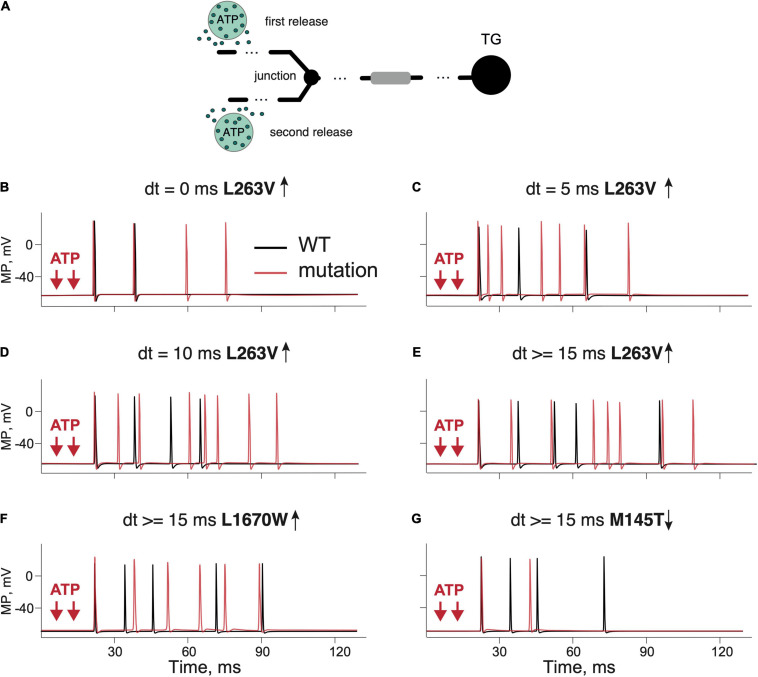
Testing the role of branching and multiple ATP release events in firing of the WT and mutant Aδ-fibers. **(A)** Schematic presentation of a dendritic tree with two branches and two ATP release events. **(B)** Firing activity of the L263V Na_V_1.1 mutant (red line) and WT (Kahlig et al., 2008) fiber (black line) with simultaneous ATP release events. **(C)** The L263V mutant (red line) and WT (Kahlig et al., 2008) fiber (black line) with ATP release with an interval of 5 ms. **(D)** The L263V mutant (red line) and WT (Kahlig et al., 2008) fiber (black line) with an 10-ms interval. **(E)** The L263V mutant (red line) and WT (Kahlig et al., 2008) fiber (black line) with an 15-ms interval. **(F)** The L1670W (red line) and WT (Dhifallah et al., 2018) fiber (black line) with an interval of 15 ms. **(G)** Firing in the loss-of-function mutant M145T (red line) and WT (Mantegazza et al., 2005) fiber (black line) with an ATP release interval of 15 ms. Note that branching and multiple ATP release increased the firing in the case of a gain-of function but reduced in the case of a loss-of-function. ATP signaling is limited by partial hydrolysis.

As it is known that meningeal afferents are branched (Schueler et al., 2014; Suleimanova et al., 2020), we next modeled the trigeminal nerves with two branches and two ATP release events activating these separate nerve branches (Figure 4A). Such model allowed us to explore the role of simultaneous and shifted in time activation of distinct branches and whether this activity may propagate to higher pain centers. We found that in case of the L263V mutation, two simultaneous ATP release events did not change the outcome number of spikes coming to TG (Figure 4B red line). However, the final number of spikes was increased by adding a second ATP release with the time interval being 5 ms (Figure 4C). With an interval of 10 ms, the number of repetitive spikes further increased from four in WT to eight spikes in L263V (Figure 4D). Similar increase was observed with interval 15 ms (Figure 4E). Then, for modeling of other mutant channels, we used a 15-ms interval between ATP release events since a further increase of the interval in the range of 15–100 ms did not change the number of produced spikes. We compared the firing for FHM3 gain-of-function mutations L263V and L1670W, and loss-of-function mutation M145T with the respective WTs. The latter, even modeled from distinct experimental studies (Mantegazza et al., 2005; Kahlig et al., 2008; Dhifallah et al., 2018) generated similar (five for L263V and L1670W and four for M145T, see Figures 4E–G) repetitive spikes. We found that the mutation L263V was much more effective in promote firing (eight spikes, Figure 4E red line) than WT (five spikes, Figure 4E black line). Firing in case of the L1670W mutation was only slightly higher than for WT (Figure 4F red line, WT–black line). Instead, the M145T mutation led to a dramatically reduced firing (Figure 4G). Thus, FHM3 mutations further increased spike frequency activated by multiple ATP release sites on branched Aδ-fibers. The branching of the single axon enhanced the spiking activity, only when it was combined with asynchronous ATP release (Figure 4) because spikes from different branches were efficiently summarized in the primary afferents.

As the concentration of extracellular ATP can vary in the neuro-immune synapse due to expression profile and location of the powerful ATP-degrading ectoenzymes (Yegutkin, 2008), we next explored, in a branched model, the role of different concentrations of ATP in generation of a single spike and on repetitive firing in the most prominent mutant L263V. The dependence of a single-spike probability from concentration of ATP and the role of ATP hydrolysis is shown in Supplementary Figure 4. In the range of the tested concentrations, the mutant L263V had a higher probability of the spike generation, with a most visible difference at lower ATP concentrations (Supplementary Figure 4A, exemplified in Supplementary Figure 4C). In case of a lack of ATP hydrolysis, the difference at low ATP concentrations was more noticeable (Supplementary Figure 4B). The repetitive firing was less sensitive to the absence or presence of ATP hydrolysis (Supplementary Figures 4G,H).

### 5-HT Induced Activation of WT and FHM3 Aδ-Fibers

5-HT is the major neurotransmitter released from meningeal mast cells that can powerfully activate peripheral trigeminal nerve terminals through ligand-gated 5-HT3 receptors (Kilinc et al., 2017; Koroleva et al., 2019). Therefore, we conducted modeling experiments (two branches, two release sites) simulating 5-HT release from mast cells and its action on 5-HT3 receptor in Aδ-fibers meningeal afferents.

**FIGURE 5:**
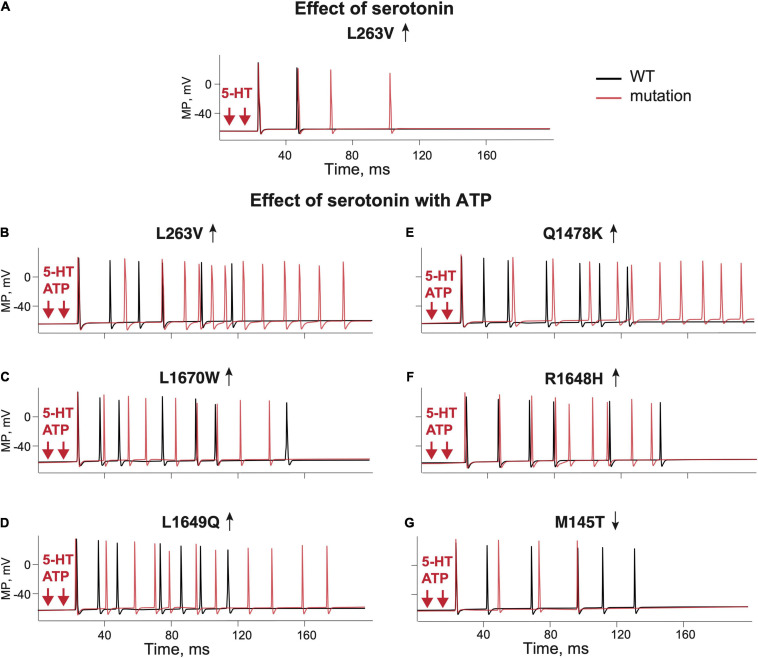
The role of 5-HT and combination of ATP and 5-HT release events in firing of the WT and Na_V_1.1 mutant branching Aδ-fibers. **(A)** Firing of the WT (Kahlig et al., 2008) fibers (black line) and the L263V mutant (red line) activated by two 5-HT release events. **(B)** WT (Kahlig et al., 2008) fibers (black line) and the L263V mutant (red line) activated by ATP + 5-HT release events. **(C)** The L1670W mutant fiber (red line) and WT (Dhifallah et al., 2018) fiber (black line) activated by ATP + 5-HT release events. **(D)** The L1649Q mutant fiber (red line) and WT (Cestele et al., 2008) fiber (black line) activated by ATP + 5-HT release events. **(E)** The Q1478K mutant fiber (red line) and WT (Cestele et al., 2013) fiber (black line) activated by ATP + 5-HT release events. **(F)** The R1648H mutant (red line) and WT (Kahlig et al., 2006) fiber (black line) associated with childhood epilepsy gain-of-function of Na_V_1.1. **(G)** The M145T Na_V_1.1 mutant (red line) and WT (Mantegazza et al., 2005) fiber (black line) associated with a loss-of-function mutation of Na_V_1.1. ATP signaling is limited by partial hydrolysis and 5-HT signals are reduced by specific uptake.

Like with ATP, we found that the excitatory action of 5-HT was twice more pronounced with the L263V mutation than with the respective WT (Figure 5A L263V–red line, WT–black line). The dependence of a single-spike probability from a concentration of 5-HT in basal conditions or after removal of 5-HT upate for the mutant L263V resembled, in some tests, that of ATP (Supplementary Figures 4D,E). In the example shown in Supplementary Figure 4F, a concentration of 5-HT as low as 0.6 μM was already able to generate a single spike in the mutant L263V, whereas the same concentration of neurotransmitter in the WT induced only a small receptor potential. We found also an increased repetitive firing with higher 5-HT concentrations for mutant L263V; in particular, when we switched from 2 to 10 μM 5-HT, not only the number of spikes but also the firing frequency rose (Supplementary Figures 5E,F). Interestingly, for mutant L263V, increasing the concentration of 5-HT in the micromolar range had a stronger effect on repetitive firing than similar changes in the concentration of ATP (Supplementary Figures 5B,F vs. Supplementary Figures 5A,D).

As ATP and 5-HT can be co-released during migraine events in the meninges (Suleimanova et al., 2020), we next modeled the simultaneous action of ATP and 5-HT. The combined activation of P2X3 and 5-HT3 receptors in WT produced more spiking activity than ATP or 5-HT alone (Figures 5B–G black lines). Then, we extended our approach to compare the firing of *SCN1A* gain- and loss-of-function mutations with their respective control WTs (Figures 5B–G red lines). With simultaneous release of ATP and 5-HT, the neuronal firing of WTs was similar (six spikes for L263V, R1648H, M145T, and seven spikes for Q1478K, and L1649Q, Figures 5B-G). Again, like in previous modeling conditions, the most pronounced spiking activity was observed with the FHM3 mutations L263V (Figure 5B), Q1478K (Figure 5E), and L1649Q (Figure 5D). The R1648H mutation (Figure 5F) that is associated with childhood epilepsy produced less spikes than the L263V mutation but more than WT.

Thus, the updated Aδ-fiber model with simultaneous action of 5-HT and ATP on the branched trigeminal nerve dramatically increased the spiking activity for the FHM3 mutations.

### Modeling the Whole Nerve Activity

Finally, to explore the role of Na_V_1.1 channels in a more physiological environment, we modeled a WT whole nerve comprising five Aδ-fibers, five C-fibers, and ten inactive (ATP- and 5-HT-insensitive) fibers (schematically presented in Supplementary Figure 6, left). In this case, to validate the modeling results with experimental results from rodent meningeal nerves comprised of Aδ- and C-fibers in trigeminal nerves (Levy et al., 2007; Haanes and Edvinsson, 2019), we added simulated C-fibers to the model of the whole nerve, although they do not express Na_V_1.1 channels, while they express P2X3 and 5-HT receptors (Zeitz et al., 2002; Yegutkin et al., 2016; Kilinc et al., 2017). Details of the C-fiber model are presented in Supplementary Figure 6. We added co-expression of P2X2 and P2X3 receptors to the whole nerve model as they are naturally expressed in trigeminal neurons of rodents (Simonetti et al., 2006). A ratio of P2X3/P2X2 was set to 75/25%, since we found in our previous study (Suleimanova et al., 2020) that this ratio closely reproduced the experimental data. The current simulated data for the whole nerve were validated with our previously published experimental results on the activity of ATP and 5-HT in mouse meningeal afferents (Koroleva et al., 2019).

**FIGURE 6:**
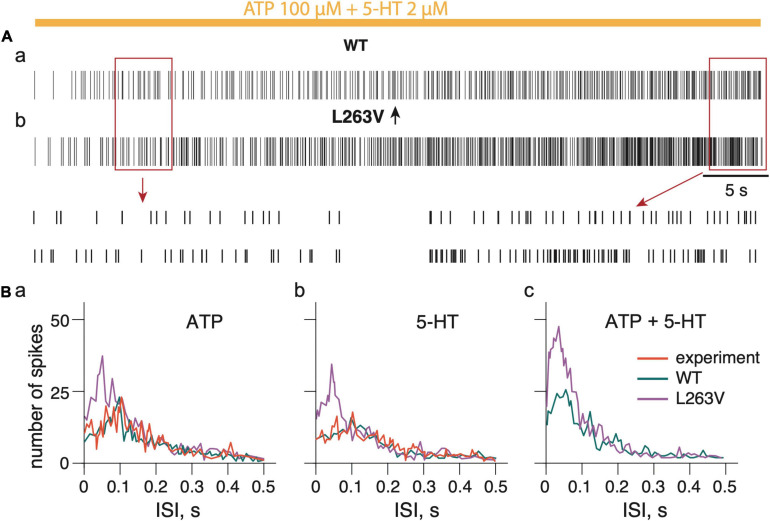
Firing activity of whole trigeminal meningeal nerve induced by persistent ATP plus 5-HT. **(Aa)** Firing activity of Na_V_1.1 WT nerve induced by combined ATP (100 μM) and 5-HT (2 μM) action. **(Ab)** Firing activity induced by combined ATP (100 μM) and 5-HT (2 μM) action on nerve with fibers expressing L263V Na_V_1.1 mutant channels. Notice details of fitting in insets. **(Ba)** The distribution of the inter-spike intervals (ISI) during action of ATP, 5-HT **(Bb)** and combined ATP + 5-HT action **(Bc).** ATP signaling is limited by partial hydrolysis and 5-HT signals are reduced by specific uptake.

For validation, we compared the inter-spike intervals (ISI) (Figure 6B) obtained from experimental data when the spiking activity in meningeal afferents was induced by 100 μM ATP (Koroleva et al., 2019) with the simulated WT model neuronal activity in afferents, also induced by 100 μM ATP (Supplementary Figure 6Aa). Likewise, we also modeled the action of 2 μM 5-HT that was also compared with experimental data with 5-HT (Koroleva et al., 2019; Supplementary Figure 6Ba). During the simulation experiments, ATP application triggered spiking which spectral profile was almost the same as in the experimental approach (Figure 6Ba). The *p*-value of 0.104 in the K-S test indicates the high similarity of the model and experimental results with ATP application. Likewise, with 5-HT, the model approach reproduced the data from the experiment (Figure 6Bb). The *p*-value of 0.128 in the K-S test indicates the high similarity of the model and experimental spikes distribution.

To reproduce the role of the FHM3 mutation in the activity of the whole nerve, we selected the L263V mutation as it produced more spikes than any other tested FHM3 mutations. In contrast to WT (Figure 6Aa), the model of the nerve with the Aδ-fiber with the L263V mutation showed a much higher neuronal activity with ATP (Figure 6Ba and Supplementary Figure 6Ab), with 5-HT (Figure 6Bb and Supplementary Figure 6Bb), and with co-application of ATP together with 5-HT (Figures 6Ab,Bc). Notably, with the L263V mutation most spikes were distributed with frequency higher than 10 Hz (ISI 0.1s).

Thus, with a FHM3 mutation an intensive spiking activity was produced in a whole nerve model with shorter intervals which suggested a more powerful nociceptive signaling.

## Discussion

The main result of this study is to provide a mechanistical explanation of peripheral mechanisms of enhanced nociceptive firing activity in trigeminal neurons and the whole nerve when *in silico* modeling the effect of FHM3 mutations. Our computational approach provides a scientific framework for the underlying molecular mechanisms of trigeminal nociceptive firing implicated in this migraine subtype. Our *in silico* approach allowed us to correct (“virtually treat”) the abnormal voltage characteristics of mutated channels that resulted in a significant reduction of nociceptive firing. Thus, our data suggest that compounds affecting Na_V_1.1 channels in Aδ-fibers of peripheral nerves in a mutation-specific manner, may be a promising avenue for novel type analgesic anti-migraine therapy.

### Role of Na_V_1.1 Channels in the Activation of Aδ-Fibers

Peripheral sensory nerves express a wide range of sodium channels needed for the generation and propagation of nociceptive spikes evoked by mechanical or chemical stimulation of nerve terminals (Basbaum and Woolf, 1999; Julius and Basbaum, 2001; Giniatullin, 2020). Among other Na_V_ subtypes, the Na_V_1.1 sodium channel subtype is highly expressed in Aδ-fibers of peripheral nerves (Ho and O’Leary, 2011; Osteen et al., 2016) in addition to in central cortical interneurons (Ogiwara et al., 2007). Their abundance predicts that dysfunction of Na_V_1.1 channels in Aδ-fibers should affect the transmission of nociceptive signals in a pronounced way. As Aδ-fibers are implicated in the generation of migraine pain (Melo-Carrillo et al., 2017; Haanes and Edvinsson, 2019), this provides a rationale for how pain signals are generated in trigeminal nerves in meninges where migraine pain is generated (Moskowitz, 2008; Messlinger, 2009; Zakharov et al., 2015).

### Trigeminal Neuron Firing in Na_V_1.1 Channels With FHM3 Mutations

Familial hemiplegic migraine type 3 (FHM3) is caused by gain-of-function mutations in the *SCN1A* gene that encodes the α1 subunit of voltage-gated Na_V_1.1 sodium channels (Castro et al., 2009). Therefore, in our model, we implemented and tested the functional role of different gain-of-function mutations (L1670W, L263V, L1649Q, and Q1478K), which were previously shown as a genetic cause of disease in patients with FHM3 (Kahlig et al., 2008; Dhifallah et al., 2018). For comparison (as a “negative control”), we also modeled nociceptive firing in trigeminal neurons with the loss-of-function mutation (M145T) in the same α1 subunit of Na_V_1.1 channels (Mantegazza et al., 2005). The strongest increase of nociceptive firing, a predictive of severe migraine pain, was observed for the L263V mutation, likely due to the increased “window current” (Figure 2Aa) because of the shift of the activation in the voltage-dependence curve to lower voltages and the inactivation curve to higher voltages comparing to WT Na_V_1.1 channels (Kahlig et al., 2008). Although, experimentally, a clear trend, albeit not significant, for increase of voltage dependence of activation (V_1__/__2_ = −21 mV for WT vs. V_1__/__2_ = −25 mV for the L263V mutation) was reported by Kahlig et al. (2008), the mutation has a clear effect in our model (Figure 2A). It is worth noting that this property was detectable only for the L263V mutant so not with the other tested mutants (Figure 2). Our L263V finding maybe not that surprising as even a small shift in activation parameters may provide a sufficiently strong functional effect on excitability, especially near threshold voltage levels when nerve terminals are activated by physiologically relevant low levels of endogenous agonists. Consistent with this, we showed that mutant L263V required less ATP or 5-HT for induction of spiking activity (Supplementary Figure 4C), which may translate to a lower pain threshold. Of note, recently, it has been shown in a knock-in mouse model that this mutation facilitated spontaneous cortical spreading depolarization (CSD) events, the likely underlying cause of the migraine aura (Jansen et al., 2020a). However, the effect of the mutation in peripheral trigeminal neurons has not been investigated. Moreover, the observation that mutated Na_V_1.1 channels can facilitate spontaneous CSD events may also have relevance to, for instance, stroke as relative peri-infarct depolarizations (PID) known to circle around the infarct core (Sukhotinsky et al., 2010), when occurring more easily, may lead to an increase in the infarct size and worse stroke outcome. Although, this has not been demonstrated in the case of FHM3, it was shown that in knock-in mice expressing Ca_V_2.1 calcium channels with a FHM1 mutation in the α1 subunit (van den Maagdenberg et al., 2004), which results in a gain-of-function and hyperexcitability phenotype (Ferrari et al., 2015), the number of PIDs was greater and the infarct size was larger when infarcts were introduced experimentally (Eikermann-Haerter et al., 2012). A hyperexcitability phenotype in FHM3 is also suggested from overexpression studies in neurons (Cestele et al., 2008; Dhifallah et al., 2018). In the case of the L1670W mutant, when overexpressed in mouse neocortical neurons, a faster recovery of the mutated Na_V_1.1 channel activity was observed (Dhifallah et al., 2018). Also, for the Q1478K mutant, when expressed in rat neocortical neurons, the gain-of-function leads to hyperexcitability, which, remarkably, was self-limiting meaning that for only a short time high-frequency neuronal discharges were maintained before a depolarizing block occurred (Cestele et al., 2008).

As trigger for receptor potentials, we used ATP and 5-HT, which are among the most powerful agonists of peripheral nociception in meningeal afferents (Yegutkin et al., 2016; Kilinc et al., 2017; Koroleva et al., 2019). The respective P2X3 and 5-HT3 receptors are expressed in Aδ-fibers (Ford, 2012; Kilinc et al., 2017; Sato et al., 2018). For all gain-of-function mutants, which show an increased persistent sodium current (L263V, L1670W, L1649Q, and Q1478K), we found, in our modeling, the activation of Aδ-fibers by ATP or 5-HT was enhanced.

This may explain why migraine pain develops in carriers of *SCN1A* gain-of-function mutations. In contrast, the loss-of-function M145T mutation (Mantegazza et al., 2005), which is associated with febrile seizures dramatically reduce spiking activity of peripheral Aδ-fibers of the trigeminal nerve. The finding of opposite functional outcomes on neuronal activity of the Na_V_1.1 mutations depending on whether they cause FHM3 or childhood epilepsy indicates that the modeling is a useful discriminating tool to predict disease outcome.

### Role of Factors Amplifying the Effect of *SCN1A* Mutations

Our mathematical model allows a “knock down” or artificial induction of a Na_V_1 channel subtype that can be used to explore molecular mechanisms that are challenging to achieve in an experimental setting. Moreover, our model allowed dissection in a stepwise approach the contribution of several of these factors, which is highly relevant as they can potentially modify the nociceptive effect of Na_V_1.1 mutations.

As a first step, we “knocked down” the contribution of the Na_V_1.6 subtype to leave only the Na_V_1.1 subtype to study its putative role in nociceptive signaling. Subsequently, we added the Na_V_1.6 to find out that Na_V_1.6, which is naturally accompanying Na_V_1.1, essentially supports the pro-nociceptive role of Na_V_1.1 in nerve terminals with a FHM3 *SCN1A* mutation.

For the second step, we added a tree structure of nerve branches to reproduce the composition of meningeal afferents more realistically, as shown experimentally (Schueler et al., 2014; Suleimanova et al., 2020) or in model (Barkai et al., 2020). The branching of a single axon combined with multiple ATP and 5-HT release events from mast cells contacting different branches can largely increase the probably of repetitive firing (Suleimanova et al., 2020). A similar amplifying role of branching was shown in the current study (Figures 4, 5). Furthermore, the slow, due to high impedance (Goldstein et al., 2019; Barkai et al., 2020) ATP/5-HT-induced receptor potential in the fine nerve terminal can initiate the persistent current through slowly inactivating Na_V_1.1. and Na_V_1.8 channels. A contributing role of Na_V_1.8 channels to the multiple firing of primary afferents was shown previously in our model of the neuro-immune synapse in meninges (Suleimanova et al., 2020). Moreover, recently we found ATP-gated (but not 5-HT-induced) nociceptive firing not only at peripheral nerve terminals but also in more central parts of trigeminal nerve fibers in rat meninges (Gafurov et al., 2021). The latter suggests that ATP can activate nerve fibers not only at the end points of an axon but also along the fiber and that this probability is higher for ATP than for 5-HT. These factors could be essential for the increased agonist-induced multiple firing of meningeal afferents. Together, our study demonstrated that branching, multiple asynchronous ATP/5-HT release events together with persistent sodium Na_V_1.1 and the presence of Na_V_1.8 currents collectively enhance repetitive nociceptive firing in Aδ-fibers, most notable in FHM3-associated mutants.

For the third step, we conducted modeling experiments with 5-HT, which is a major nociceptive mediator in meninges, likely released from mast cells during migraine attacks, and that can strongly activate meningeal fibers through 5-HT3 receptors (Kilinc et al., 2017; Koroleva et al., 2019). However, unlike seen for the combined action of ATP and 5-HT, which induces very pronounced and long-term spiking activity, 5-HT alone induced firing that was not as strong as seen with ATP, likely due to a lower amplitude of 5-HT3 receptor-mediated generator potential at nerve terminals.

Notably, the concentration of ATP and 5-HT released within the meningeal neuro-immune synapse has a different time profile (Suleimanova et al., 2020). In the case of ATP, the time course is primarily determined by enzymatic hydrolysis of this endogenous purinergic transmitter by numerous ectoATPases (Yegutkin, 2008). Therefore, ATP-induced excitation depends on the spatial distribution of these enzymes in the neuro-immune synapse. Any mismatch between the ATP release site and the presence of ectoATPases in the synapse would increase the excitatory nociceptive action of this purinergic transmitter due to reduced transmitter hydrolysis as shown in the current study for the case of absence of ectoATPases activity in the meningeal synapse. Interestingly, in experimental migraine-like conditions induced by the migraine mediator CGRP, the level of endogenous ATP in the rat meninges is significantly enhanced (Yegutkin et al., 2016) by increasing purinergic drive for nociceptive excitation.

In contrast to ATP, released 5-HT is removed from the neuro-immune synapse by a relatively slow uptake (Suleimanova et al., 2020). This is specific for the 5-HT transport system and can be blocked by selective serotonin reuptake inhibitors (SSRIs), commonly used in patients with depression. The inhibition of specific transporters can increase the level of serotonin in the extracellular space and potentially amplify the pro-nociceptive action of 5-HT in meninges. In opposite, an increased expression of serotonin transporters is expected to provide an anti-nociceptive effect, specifically when switching off the serotonergic drive for excitation of nerve terminals via 5-HT3 receptors (Kilinc et al., 2017).

We also explored the effect of ATP and 5-HT concentrations as they could vary in migraine-relevant conditions. In the meningeal neuro-immune synapse, the concentration and the time profile for these two potential triggers of nociceptive firing is very different as determined by distinct elimination mechanisms such as enzymatic degradation and uptake, respectively (Yegutkin, 2008; Wood et al., 2014; Suleimanova et al., 2020). Indeed, we found that the shift from basal conditions with partial ATP hydrolysis and functional 5-HT uptake (Supplementary Figures 4G,I) to complete prevention of ATP hydrolysis and removal of 5-HT uptake (Supplementary Figures 4H,J) significantly increased repetitive firing of meningeal afferents. Consistent with this, the number of spikes was also increased when enhancing the concentration of both these algogens from 2 to 10 μM, but it was more noticeable with 5-HT (Supplementary Figures 4C–F).

In our next step, we simulated the more realistic multi-fiber complex nerve model to compare the computational outcome with results from experiments performed previously (Koroleva et al., 2019). Notably, in the model of the whole nerve, ATP and 5-HT produced a powerful long-lasting firing in in analogy to results observed with firing that was induced by ATP and 5-HT in the experimental condition in mouse meninges. Most relevant to this study, nociceptive spiking of the whole nerve activity largely increased when a FHM3 mutation was introduced in the model of the Aδ-fibers, indicating that our model properly reproduces an enhanced nociceptive firing in FHM3.

### Virtual Treatment *in silico* of Carriers of *SCN1A* Mutations

**FIGURE 7:**
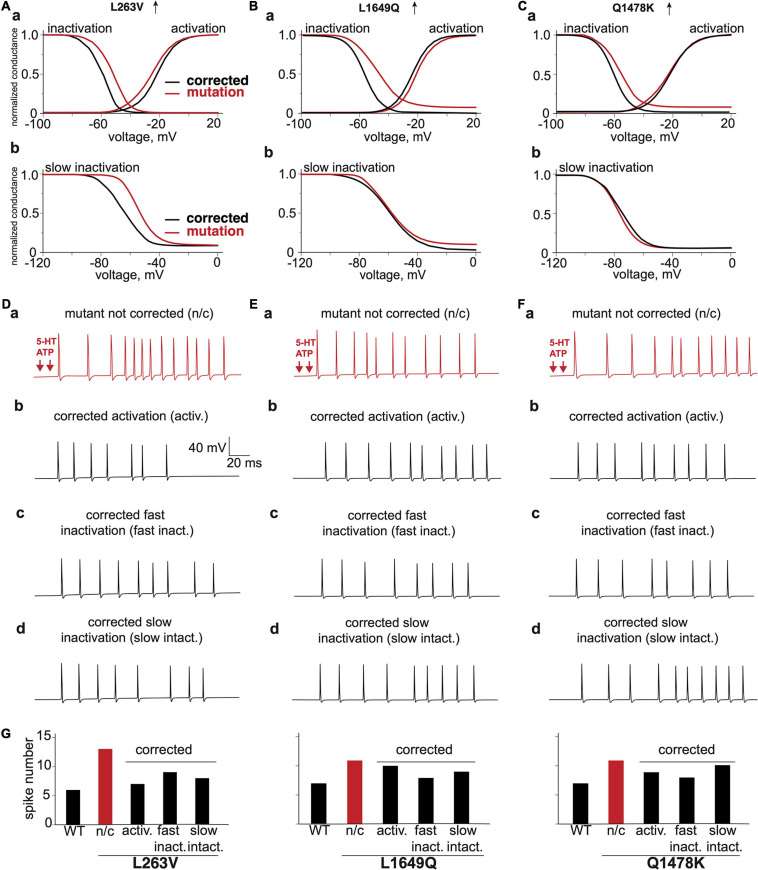
The virtual correction of the abnormal phenotype via modification of voltage characteristics of mutated channels. The comparison of voltage dependence of corrected activation, fast inactivation and mutation **(Aa)** L263V, **(Ba)** L1649Q, **(Ca)** Q1478K. The comparison of voltage dependence of corrected slow inactivation and mutation **(Ab)** L263V, **(Bb)** L1649Q, and **(Cb)** Q1478K. **(Da)** L263V mutant fiber activated by ATP + 5-HT release events, **(Db)** with corrected activation, **(Dc)** with corrected fast inactivation, **(Dd)** with corrected slow inactivation. **(Ea)** L1649Q mutant fiber activated by ATP + 5-HT release events, **(Eb)** with corrected activation, **(Ec)** with corrected fast inactivation, **(Ed)** with corrected slow inactivation. **(Fa)** Q1478K mutant fiber activated by ATP + 5-HT release events, **(Fb)** with corrected activation, **(Fc)** with corrected fast inactivation, **(Fd)** with corrected slow inactivation. **(G)** The spike number of WT fiber and L263V, L1649Q, and Q1478K mutations with correction of activation and inactivation. ATP signaling is limited by partial hydrolysis and 5-HT signals are reduced by specific uptake.

Since different mutations exhibit distinct changes in the voltage dependence such as activation, fast or slow inactivation (Figure 2), we next set out to correct *in silico* the pathological phenotype. To this end, we used as an indicator of “treatment efficiency” the reduction of spiking in meningeal afferents and compared the result with the firing in the WT model (Figure 7G).

We observed for mutant L263V that its nociceptive phenotype was associated with abnormal changes of activation, fast and slow inactivation (Figures 7Aa,Ab,Da). The best improvement was obtained with corrected activation (Figure 7Db), since mutant L263V is activated at a more negative voltage than WT that showed in Figures 2Aa, 7Aa as a shift of the voltage-dependence activation curve to the left and shows a delayed entry into fast inactivation state. Besides, this mutation accelerated recovery, and reduced depolarizing shift in the voltage dependence of both fast and slow inactivation. However, the correction of fast and slow inactivation states in L263V less efficiently reduced spiking activity (Figures 7Dc,Dd,G). In the case of mutants L1649Q and Q1478K (Figures 7Ea,Fa), which profoundly exhibit a positive shift in fast inactivation (Figures 7Ba,Ca), the number of spikes, as expected, was most strongly decreased when we corrected the fast inactivation (Figures 7Ec,Fc,G). Since there were no significant alterations in the slow inactivation state for mutants L1649Q and Q1678K (Figures 7Bb,Cb) correction of this state did not significantly change the neuronal spiking activity (Figures 7Ed,Fd).

Our data suggest that treatment of FHM3 patients that exhibit headache as a main complaint, but due to a different Na_V_1.1 mutation, may be achieved through two distinct effects of possibly different drugs, as in some cases, the most efficient correction was based on improvement of activation (L263V), whereas in other cases, the best results was obtained with correction of the inactivation state (L1649Q and Q1478K). In addition to direct correction of the genetic defect, there might be alternative pharmacological mechanisms to limit the FHM3-associated enhanced repetitive firing in trigeminal neurons. One possibility may be the activation of potassium-mediated K_V_7/M-currents, which contribute to the stabilization of the membrane potential and can be activated by the analgesic drugs, such as paracetamol (Ray et al., 2019).

In our modeling approach, we did not simulate the outcome of changes in Na_V_1.1 in central neurons where these channels are expressed in interneurons (Favero et al., 2018; Sakkaki et al., 2020). Recently, computer modeling of various neuron types, similar to our whole nerve paradigm, was used to predict functional outcome of an FHM3 mutation on the central neuronal network of transgenic Dravet mice, in which Na_V_1.1 channels were ablated in hippocampus and cortex (Jansen et al., 2020b). One could model the role of Na_V_1.1 channels in CSD events associated with FHM3 or analogous PID in stroke to assess brain recovery after ischemia (Sukhotinsky et al., 2010). The proposed modeling approach can also actively guide the development of selective activators or inhibitors of Na_V_1.1 channels to treat diseases such as hemiplegic migraine, seizures and stroke.

In summary, our data demonstrated a long-term intensive spiking activity in meningeal afferents, with various gain-of-function mutations of sodium channels associated with FHM3 in patients. Improvement of high nociceptive activity was obtained in a mutation-specific manner, being in some cases based on correction of the activation process (Figures 7Db,Eb,Fb), whereas, in others, a better result was obtained after adjustment of both the fast and slow inactivation of mutated Na_V_1.1 channels (Figures 7Dc,Dd,Ec,Ed,Fc,Fd). Such modeling provides a new tool for the exploration of peripheral mechanisms in trigeminal pain and suggests that molecules reducing, probably in a mutation-specific manner, the excessive activity of Na_V_1.1 channels could present a novel type of analgesic therapy for patients with migraine.

## Data Availability Statement

The raw data supporting the conclusions of this article will be made available by the authors, without undue reservation.

## Author Contributions

AS and MT contributed to modeling, analysis and writing the manuscript. AMJMvdM contributed to design of the study, interpretation, writing the manuscript, and the final editing. RG contributed to the study design and supervision, writing the manuscript, and the final editing. All authors approved the final version of the manuscript.

## Conflict of Interest

The authors declare that the research was conducted in the absence of any commercial or financial relationships that could be construed as a potential conflict of interest.
