# Supplementary material for: Deciphering in silico the Role of Mutated NaV1.1 Sodium Channels in Enhancing Trigeminal Nociception in Familial Hemiplegic Migraine Type 3
Source: Front Cell Neurosci. 2021 May 31;15:644047. doi: 10.3389/fncel.2021.644047 (PMC8200561; doi:10.3389/fncel.2021.644047)
Supplement: Supplementary file 1 [file Data_Sheet_1.pdf]

Supplementary Material

Deciphering *in silico* the role of mutated $Na_V1.1$ sodium channels in enhancing trigeminal nociception in familial hemiplegic migraine type 3

Alina Suleimanova, Max Talanov, Arn M J M van den Maagdenberg and Rashid Giniatullin

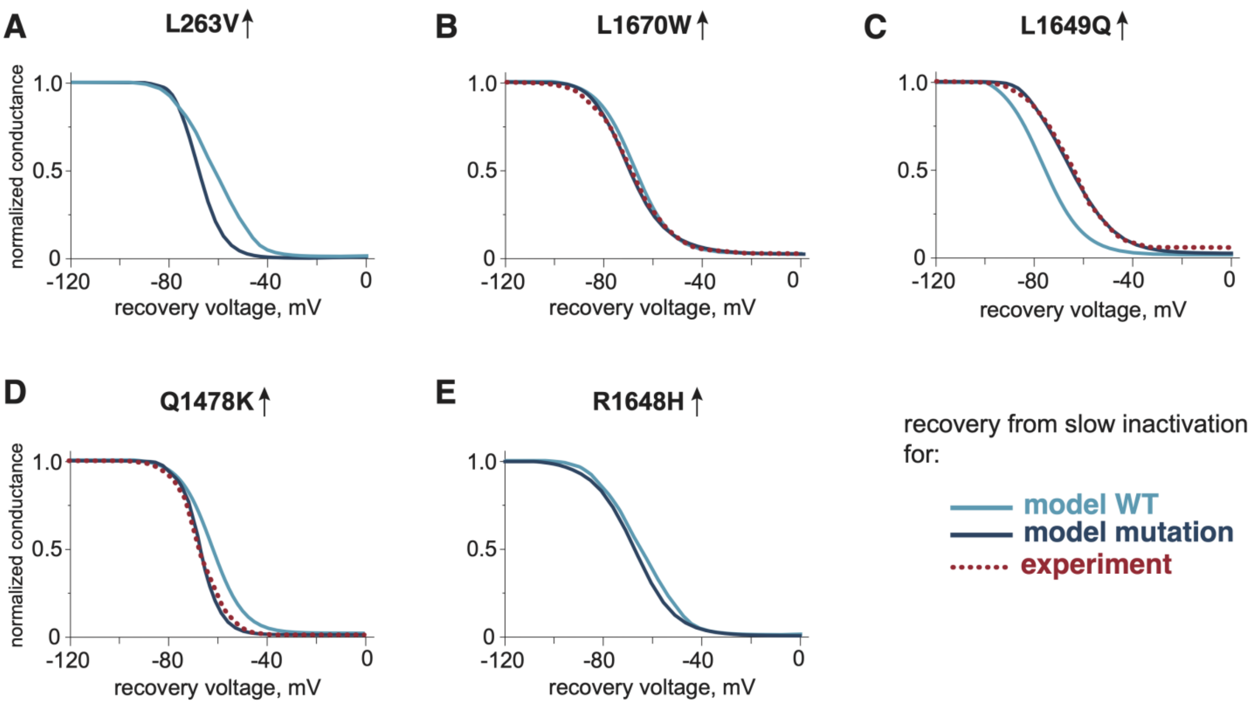

Supplementary Figure 1. The comparison of voltage dependence of recovery from slow inactivation of $Na_V1.1$ WT and gain-of-function mutants L263V**(A),** L1670W**(B),** L1649Q**(C),** Q1478K**(D),** R1648H**(E).** We compared mutations with WT which are presented in the same paper from where we used experimental data for the mutation. Kahlig et al. (2008) for the L263V mutation, Dhifallah et al. (2018) for the L1670W mutation, Cestele et al. (2008) for the L1649Q mutation, Cestele et al. (2013) for the Q1478K mutation, Kahlig et al. (2006) for the R1648H mutation, and Mantegazza et al. (2005) for the M145T mutation.

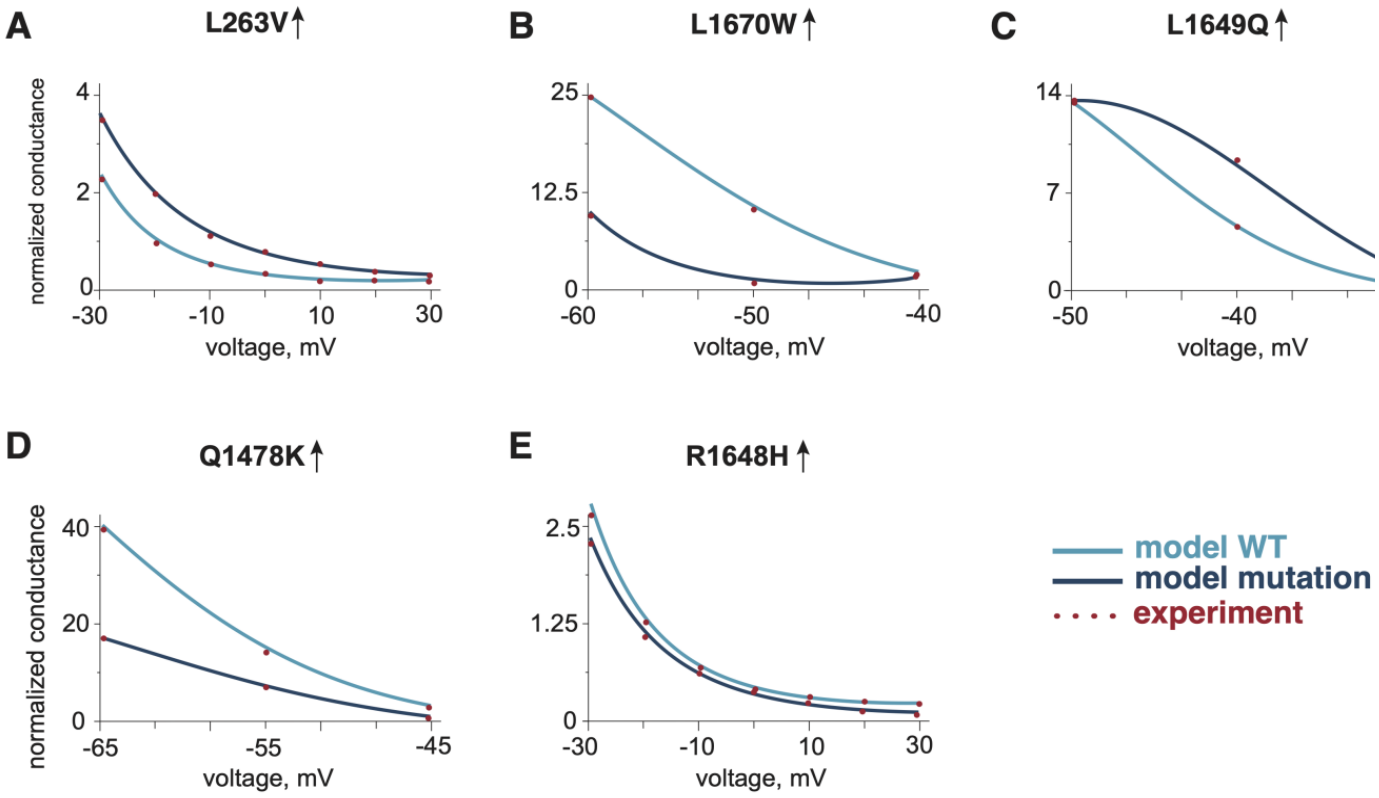

Supplementary Figure 2. The comparison of tau fast as a function of voltage of $Na_V1.1$ WT and gain-of-function mutants L263V**(A),** L1670W**(B),** L1649Q**(C),** Q1478K**(D),** R1648H**(E).** We compared mutations with WT which are presented in the same paper from where we used experimental data for the mutation. Kahlig et al. (2008) for the L263V mutation, Dhifallah et al. (2018) for the L1670W mutation, Cestele et al. (2008) for the L1649Q mutation, Cestele et al. (2013) for the Q1478K mutation, Kahlig et al. (2006) for the R1648H mutation, and Mantegazza et al. (2005) for the M145T mutation.

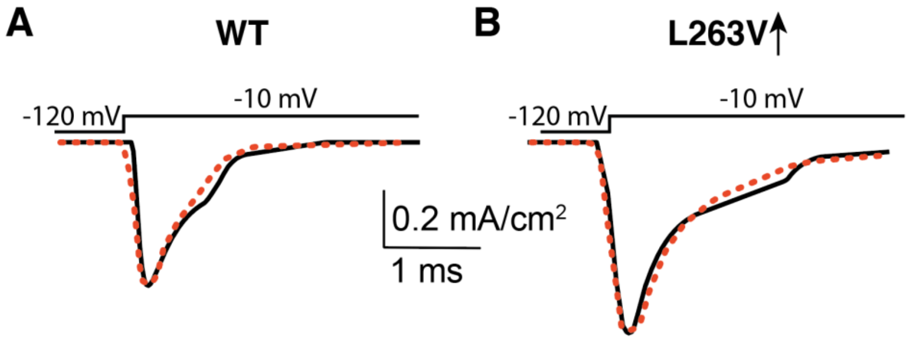

Supplementary Figure 3. The comparison of simulated sodium Nav1.1 current with experimental results. (A) The normalized to peak current of Nav1.1 WT and experimentally recorded (Kahlig et al. 2008) sodium current of Nav1.1 WT (dotted line). **(B)** The normalized current of L263V compared with experimentally recorded (Kahlig et al. 2008) sodium current of L263V (dotted line). All currents were evoked by a voltage step to -10mV. To extract experimental data we traced graph from paper in Adobe Illustrator, then, we put vectorized curve above simulated current according to time scale.

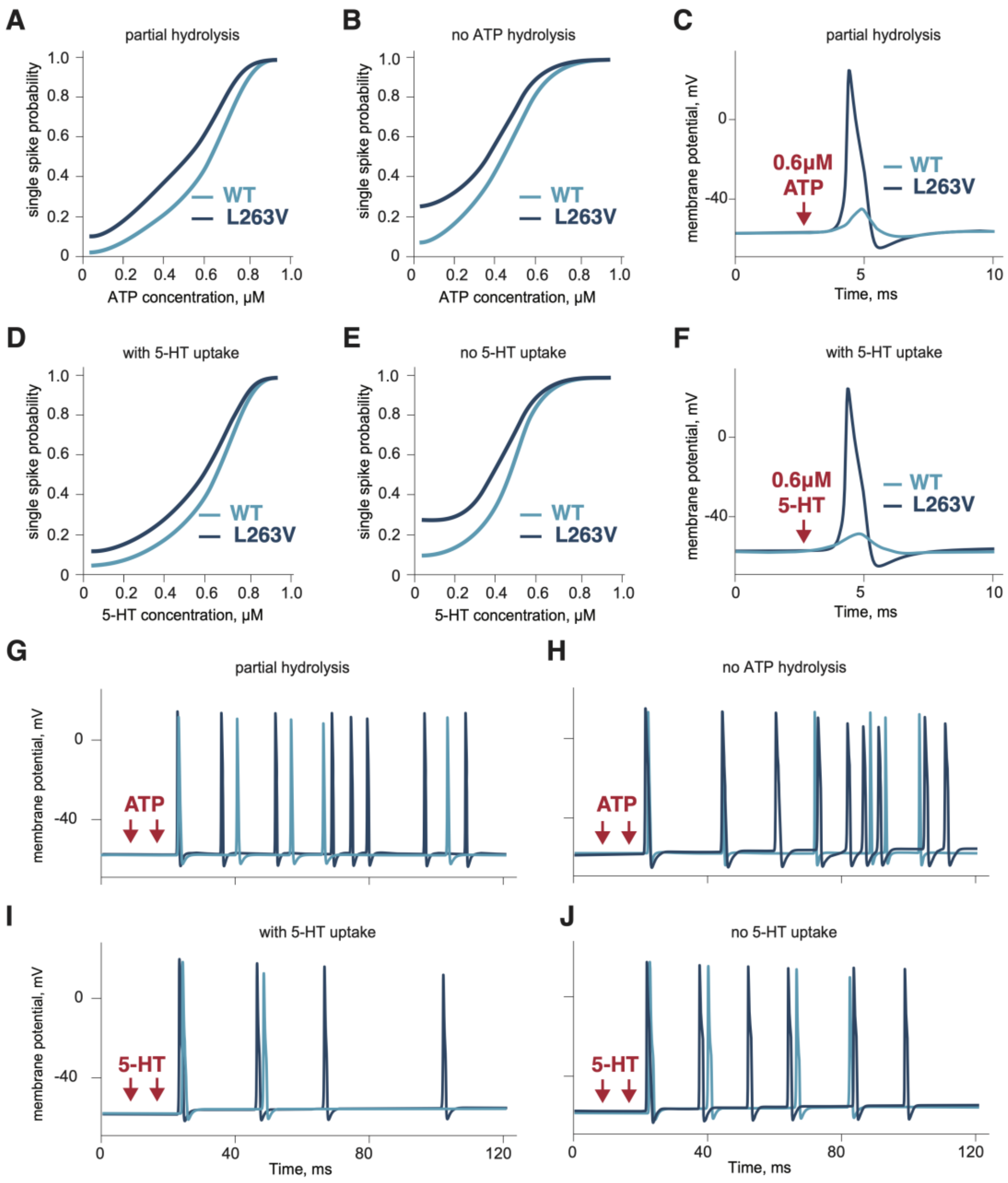

Supplementary Figure 4. The dependence of a single spike probability from concentration of ATP or 5-HT and the role of ATP hydrolysis and 5-HT uptake. (A) The dependence of ATP concentration with partial hydrolysis and single spike probability for Na_V1.1 WT and mutant L263V. **(B)** The dependence of ATP concentration without hydrolysis and single spike probability for Na_V1.1 WT and mutant L263V. **(C)** The voltage changes of the Na_V1.1 WT and mutant L263V expressing fibers activated with 0.6 µM ATP without hydrolysis with partial hydrolysis. **(D)** The dependency of 5-HT concentration with uptake and single spike probability for Na_V1.1 WT and mutant L263V. **(E)** The dependency of 5-HT concentration without uptake and single spike probability for Nav1.1 WT and mutant L263V. **(F)** The voltage of the Na_V1.1 WT and mutant L263V fibers with 0.6 µM 5-HT application with uptake. **(G)** Firing of the Na_V1.1 WT and mutant L263V fibers activated by two ATP release events with partial hydrolysis and **(H)** without hydrolysis. **(I)** Firing of the WT and mutant L263V fibers activated by two 5-HT release events with uptake and **(J)** without uptake.

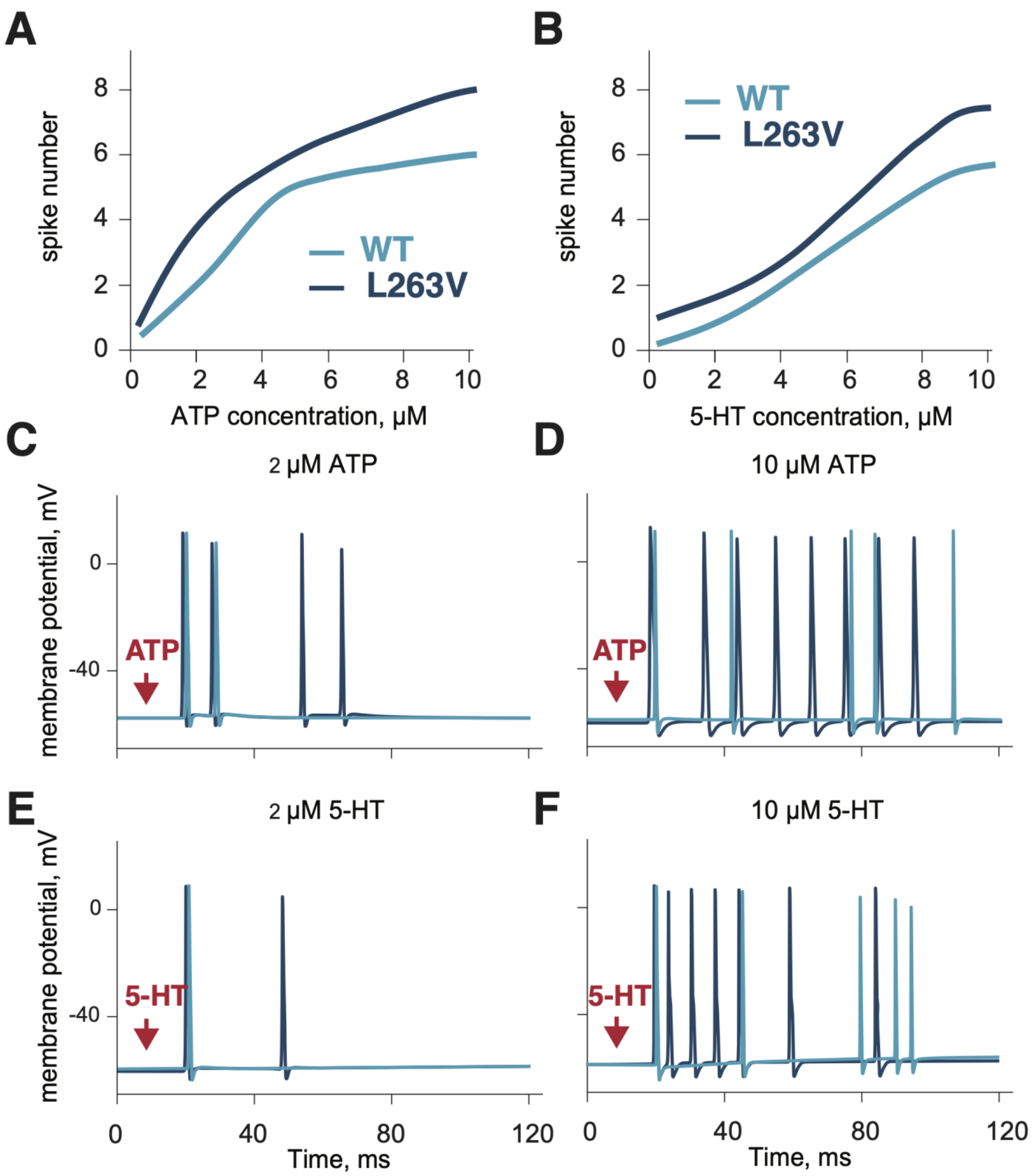

Supplementary Figure 5. The dependence of repetitive firing from ATP or 5-HT concentration. **(A)** The dependence of ATP concentration with partial hydrolysis and the number of spikes. **(B)** The dependency of 5-HT concentration with uptake and the number of spikes. (**C**) The membrane potential (in mV) of $Na_V1.1$ WT and mutant L263V fibers with 2 µM ATP and **(D)** 10 µM ATP application. **(E)** The membrane potential (in mV) of the $Na_V1.1$ WT and mutant L263V fibers with application of 2 µM 5-HT and **(F)** 10 µM 5-HT.

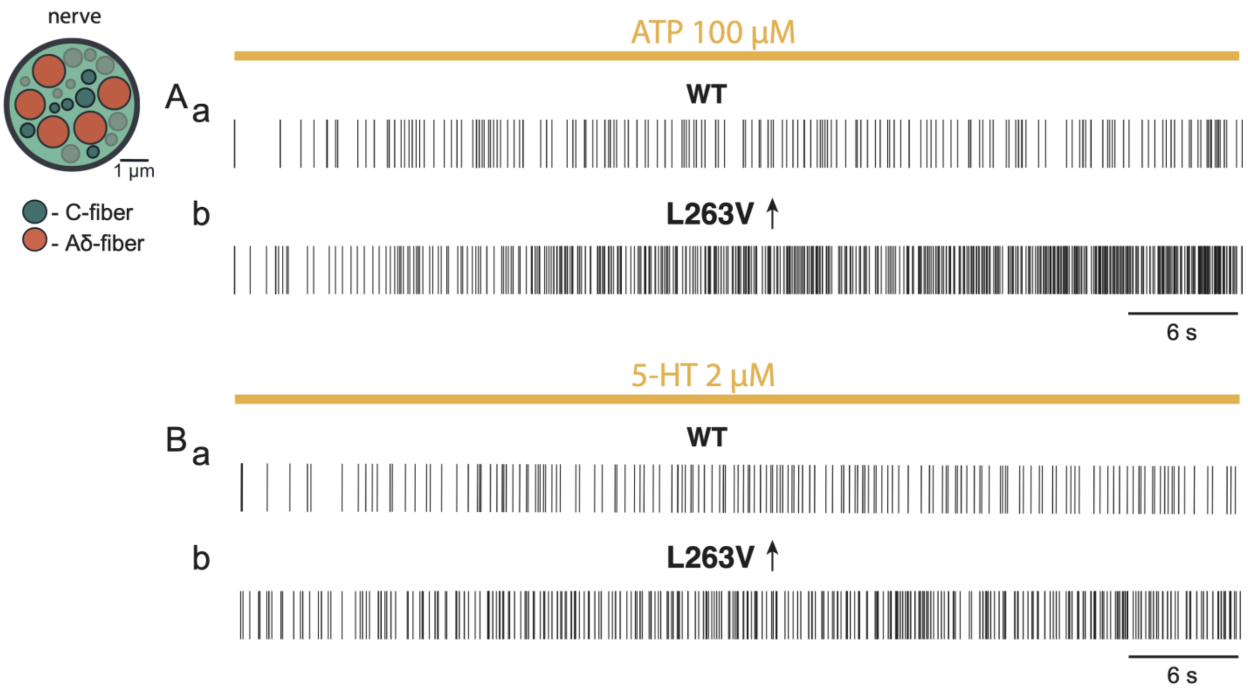

Supplementary Figure 6. The modeling of action of ATP and 5-HT on the whole trigeminal nerve (left) composed by five Aδ- and five C-fibers and 10 insensitive to ATP and 5-HT fibers. The firing activity of Na_v1.1 WT **(Aa)** and mutant L263V **(Ab)** nerve induced by 100 µM ATP. The firing activity of Na_v1.1 WT **(Ba)** and mutant L263V **(Bb)** nerve induced by 2 µM 5-HT.

To simulate the whole nerve activity, we additionally developed the model of C-fiber since these fibers, like Aδ ones, are also responding to ATP and 5-HT. The model of C-fiber has diameter 0.25 µm and two P2X3 or 5-HT3 receptors (Sokolova et al., 2006; Corradi et al., 2009) in each compartment (0.25 µm x 250 µm). The membrane capacitance was 1 µF/cm2. We activated receptors applying 1 µM ATP with partial hydrolysis and 2 µM 5-HT with uptake. These concentrations are close to EC50 of P2X3 and 5-HT3 receptors (Supplementary Table 1). For C-fiber model the following set of ion channel was used: Nav1.7 and Nav1.8 (Ho and O'Leary, 2011; Zhang et al., 2013; Balbi et al., 2017) with conductances 0.1 S/cm^2 and 0.2 S/cm^2, respectively, the densities were based on the axon model of C-fiber by Tigerholm et al. (2014) (for Na_V1.7 is 106.64 mS/cm^2, for Na_V1.8 is 242.71mS/cm^2), A-type current (Gasparini et al., 2004): Kv1 with g = 0.02 S/cm^2, Kv2 with g = 0.04 S/cm^2, Kv4 with g = 0.02 S/cm^2, calcium-activated potassium channel KCa with g = 0.001 S/cm^2 (Mandge and Manchanda, 2018), and potassium delayed rectifier KDR with g = 0.01 S/cm^2 (Tigerholm et al., 2014). Details of the model we reported previously (Suleimanova et al., 2020)

Supplementary Table 1

Morphological and functional properties of Aδ-fiber

Parameter	Value	Reference
Fiber diameter	5 µm	(West et al., 2015)
Node diameter	2 µm	(McIntyre et al., 2002)
Capacitance	2 µF/cm^2	(McIntyre et al., 2002)
Leakage conductance	0.007 S/cm^2	(McIntyre et al., 2002)
EC50 of P2X3	1.5 µM	(Sokolova et al., 2006)
EC50 of 5-HT3	2.7 ± 0.5 µM	(Corradi et al., 2009)
maximum conductance density of sodium channels		
Na_V1.1	0.35 - 0.5 S/cm^2	Model based on channel properties experimentally recorded by Kahlig et al. (2008)
Na_V1.6	0.35 - 0.5 S/cm^2	Model by Zheng et al. (2019)
Na_V1.7	0.3 S/cm^2	Model by Tigerholm et al. (2013)
Na_V1.8	0.05 S/cm^2	Model by Balbi et al. (2017)
Conductance fitted taking into account channel density (McIntyre et al., 2002) mRNA level (Ho and O’Leary, 2011), contribution to I_{Na} (Zhang et al., 2013; Pinto et al., 2008), and firing (Cestele et al., 2008; Cestele et al., 2013).		
maximum conductance density of potassium channels		
K_V1	0.04 S/cm^2	Model by Zheng et al., (2019) conductance fitted based on channel density (Zemel et al., 2018; Chien et al., 2007; McIntyre et al., 2002).
K_V3	0.04 S/cm^2	
K_V4	0.025 S/cm^2	
K_{Ca}	0.001S/cm^2	Model by Mandge and Manchanda (2018).

References

Balbi, P., Massobrio, P., and Hellgren Kotaleski, J. (2017). A single Markov-type kinetic model accounting for the macroscopic currents of all human voltage-gated sodium channel isoforms. *PLoS Comput. Biol.* 13:e1005737. doi: 10.1371/journal.pcbi.1005737

Chien L. Y., Cheng J. K., Chu D., Cheng C. F., Tsaur M. L. (2007). Reduced expression of A-type potassium channels in primary sensory neurons induces mechanical hypersensitivity. *J. Neurosci.* 27. doi: 10.1523/JNEUROSCI.0604-07.2007

Corradi, J., Gumilar, F., and Bouzat, C. (2009). Single-channel kinetic analysis for activation and desensitization of homomeric 5-HT3A receptors. *Biophys. J.* 97, 1335–1345. doi: 10.1016/j.bpj.2009.06.018

Gasparini, S., Migliore, M., and Magee, J. C. (2004). On the initiation and propagation of dendritic spikes in CA1 pyramidal neurons. *J. Neurosci.* 24, 11046–11056. doi: 10.1523/JNEUROSCI.2520-04.2004

Ho C. and O'Leary M. E. (2011). Single-cell analysis of sodium channel expression in dorsal root ganglion neurons. *Mol. Cell. Neurosci.* 46(1), 159-166. doi: 10.1016/j.mcn.2010.08.017.

Mandge, D., and Manchanda, R. (2018). A biophysically detailed computational model of bladder small DRG neuron soma. P*LoS Comput. Biol.* 14:e1006293. doi: 10.1371/journal.pcbi.1006293

McIntyre C. C., Richardson A. G., and Grill W. M. (2002). Modeling the excitability of mammalian nerve fibers: influence of afterpotentials on the recovery cycle. *J. Neurophysiol.* 87(2), 995-1006. doi: 10.1152/jn.00353.2001.

Pinto V., Derkach V.A., Safronov B.V. (2008) Role of TTX-sensitive and TTX-resistant sodium channels in Adelta- and C-fiber conduction and synaptic transmission. J Neurophysiol. 99(2):617-28. doi: 10.1152/jn.00944.2007.

Sokolova, E., Skorinkin, A., Igor, M., Agrachev, A., Nistri, A., and Giniatullin, R. (2006). Experimental and modeling studies of desensitization of P2X3 receptors. *Mol. Pharmacol.* 70, 373–382. doi: 10.1124/mol.106.023564

Tigerholm, J., Petersson, M. E., Obreja, O., Lampert, A., Carr, R., Schmelz, M., et al. (2014). Modeling activity-dependent changes of axonal spike conduction in primary afferent C-nociceptors. *J. Neurophysiol.* 111, 1721–1735. doi: 10.1152/jn.00777.2012

West S. J., Bannister K., Dickenson A. H., and Bennett D. L. (2015). Circuitry and plasticity of the dorsal horn–toward a better understanding of neuropathic pain. *Neuroscience*, 300, 254-275. doi: 10.1016/j.neuroscience.2015.05.020.

Zemel B. M., Ritter D. M., Covarrubias M., and Muqeem T. (2018). A-type Kv channels in dorsal root ganglion neurons: diversity, function, and dysfunction. *Front. Mol. Neurosci.* 11:253. doi: 10.3389/fnmol.2018.00253

Zhang M.M., Wilson M.J., Gajewiak J., Rivier J.E., Bulaj G., Olivera B.M., et al. (2013). Pharmacological fractionation of tetrodotoxin-sensitive sodium currents in rat dorsal root ganglion neurons by μ-conotoxins. *Br. J. Pharmacol.* 169(1):102-14. doi: 10.1111/bph.12119

Zheng Y., Liu P., Bai L., Trimmer J. S., Bean B. P., and Ginty D. D. (2019). Deep sequencing of somatosensory neurons reveals molecular determinants of intrinsic physiological properties. *Neuron* 103(4), 598-616. doi: 10.1016/j.neuron.2019.05.039
